# Survival Evaluation of Restorations in Pulpotomized Primary Teeth with MTA or Biodentine^®^: A Systematic Review

**DOI:** 10.3390/jcm14155501

**Published:** 2025-08-05

**Authors:** María Biedma-Perea, María Moscoso-Sánchez, María José Barra-Soto, Marcela Arenas-González, David Ribas-Pérez, Carolina Caleza-Jiménez

**Affiliations:** Pediatric Dentistry, Faculty of Dentistry, University of Seville, C/Avicena S/N, 41009 Seville, Spain; mbiedma1@us.es (M.B.-P.); mbarra@us.es (M.J.B.-S.); dribas@us.es (D.R.-P.); ccaleza@us.es (C.C.-J.)

**Keywords:** Biodentine, MTA, pulpotomy, primary teeth, restorations

## Abstract

**Objective**: Due to the increasing esthetic demand among pediatric patients and different restorative materials, we focused on analyzing which of the options of restorations may provide superior clinical outcomes. **Methods**: A systematic review was conducted according to the Preferred Reporting Items for Systematic Reviews and Meta-Analyses (PRISMA) using PubMed and Cochrane databases. **Results**: Five articles met all inclusion criteria from an initial pool of 359 articles identified in the initial search. Greater bond strength was observed when pulpotomized teeth were restored with Biodentine^®^ and resin composites compared to resin-modified glass ionomer cements (RMGICs). When comparing pulpotomies in primary teeth with MTA and Biodentine^®^, the fracture resistance values were higher in the pulpotomized teeth with Biodentine^®^ than with MTA. Additionally, following a narrative synthesis in MTA-treated teeth, a higher risk of failure was observed using RMGICs or composite instead of stainless-steel crowns (SCCs) as the final restorative material. **Conclusions**: Variables such as the type of final restoration can affect the survival of primary teeth after pulpotomy reconstruction. Regardless of the pulp material, survival with SSCs is higher, but resin composites appear to be a viable restorative material after Biodentine^®^ application.

## 1. Introduction

With the advent of various biocompatible ceramic materials [1], a paradigm shift in therapeutic pulp treatments has occurred. These materials exhibit enhanced sealing properties and notable antibacterial and antifungal activity. In addition, they can mimic human tissue function and actively stimulate the regenerative potential of adjacent biological structures. Historically, the composition of bioceramic cements has been derived from Portland cement formulations [2]. Mineral trioxide aggregate (MTA) and Biodentine^®^ have emerged as the most effective agents for pulp capping and tissue regeneration. Due to their intrinsic properties, both have demonstrated a consistent ability to promote the early formation of reparative and tertiary dentin. Their high rates of clinical and radiographic success underscore the need for further comparative studies to establish the superiority of one over the other [3]. Given these advantageous characteristics, bioceramic cements have become the materials of choice for contemporary pulp therapy, where their mechanical properties are as important to long-term outcomes as their biological compatibility. Equally important is the integrity of the adhesive interface between these biomaterials and restorative agents, as it plays a critical role in ensuring the durability and success of treatment [2,3].

In the treatment of primary teeth undergoing pulp therapy, the definitive coronal restoration is a critical determinant of overall treatment success [4], as failure of the restoration can lead to bacterial microleakage and subsequent failure of pulp therapy. Considering the importance of maintaining primary teeth until their physiological exfoliation, it is essential to employ restorative strategies that ensure both longevity and efficacy [5,6,7]. The use of durable and sealing restorative materials after pulpotomy has been shown to significantly influence clinical outcomes. For several decades, stainless-steel crowns (SSCs) have been the restoration of choice for pulpotomized primary molars. SSCs offer durable protection for structurally compromised teeth and effectively prevent marginal leakage, thereby ensuring an adequate coronal seal. However, their limited esthetic appearance and contraindications in patients with metal hypersensitivity have necessitated the exploration of alternative restorative options [8]. The increasing emphasis on esthetics, driven largely by the expectations of patients and caregivers, has led to the development of full-coverage restorations, such as prefabricated zirconia crowns, with improved visual appearance. Zirconia crowns are characterized by high compressive strength, excellent fracture and corrosion resistance, superior biocompatibility, and favorable longevity. They are manufactured from high-strength polycrystalline ceramics without a glass phase. In addition, they offer a significant esthetic advantage over traditional metal crowns. However, their application requires a more extensive preparation of the tooth, which may extend the time required to complete treatment, and they are associated with increased wear of opposing enamel surfaces [9].

Owing to their properties’ esthetic appearance, resin composites represent a valuable material in restorative dentistry. However, intrinsic characteristics, such as polymerization shrinkage, must be considered, which can generate internal stress within the tooth structure, leading to cusp deflection and restoration failure [9]. The development of new generations of resin composites, specifically bulk fill materials that enable cavity restoration in a single increment, has contributed to the reduction of polymerization stress and chair time, both of which are crucial in pediatric dental care. Furthermore, these resins satisfy the increasing esthetic demands of patients and caregivers without requiring special or invasive tooth preparations [10]. In this context, the adhesive interface between the biomaterial and the restoration must be considered, as it plays a critical role in the clinical outcome of pulp therapy. A reliable adhesive bond enables clinicians to determine the most effective combination of materials to achieve predictable long-term success [3]. The evolution of adhesive systems has resulted in the development of various generations, and studies have shown that modern adhesives exhibit uniform interfaces free of voids, cracks, or delamination, which are indicative of a stable and effective bond [3].

Conversely, resin composites are characterized by polymerization shrinkage and a high modulus of elasticity, which can generate internal stress under occlusal load, compromising restoration longevity [11,12]. In search of alternative restorative materials, studies have increasingly highlighted the use of glass ionomer cements (GICs), which exhibit an elastic modulus similar to that of dentin and possess the additional benefit of fluoride release, contributing to the prevention of demineralization of adjacent tooth surfaces.

However, GICs exhibit certain limitations, particularly in terms of esthetics, as their translucency and limited color range result in suboptimal visual outcomes. Furthermore, their relatively low abrasion resistance contributes to their progressive surface roughness over time [12]. To enhance the physical and mechanical properties of GICs, resin-modified glass ionomer cements (RMGICs) were introduced. These formulations incorporate light-curable resin monomers, which significantly improve the mechanical performance of GICs [13]. Additional modifications, such as the incorporation of nanoparticles and the application of thermal light-curing techniques, are expected to enhance the mechanical resilience of GIC-based restorations [14,15,16,17]. However, the current clinical data on the performance and longevity of these newly developed materials are limited. In addition to the use of full-coverage crowns, a wide array of restorative approaches for pulpotomized teeth is available in pediatric dentistry. Given the critical importance of ensuring resistance and structural integrity until physiological tooth exfoliation, we evaluated which restorative modalities offer superior long-term results.

## 2. Materials and Methods

### 2.1. Protocol and Registration

This systematic review was conducted in accordance with the Preferred Reporting Items for Systematic Reviews and Meta-Analyses (PRISMA) 2020 guidelines [18], and was prospectively registered in the International Prospective Register of Systematic Reviews (PROSPERO) under the protocol number CRD42024593207.

### 2.2. Review Question

The central research question of this systematic review was the following: Which restorative options instead of preformed crowns offer long-term mechanical strength in primary teeth treated with pulpotomies using bioceramic materials? This question was formulated using the PICO framework [19], which comprises the following components:

(1) Population: Primary teeth undergoing pulpotomy with mineral trioxide aggregate (MTA) or Biodentine^®^;
(2) Intervention: Coronal restoration of the pulpotomized tooth;
(3) Comparators: Various restorative materials used post-pulpotomy;
(4) Outcome: Clinical success of restorative treatment.

### 2.3. Eligibility Criteria

Inclusion and exclusion criteria were established according to the Strength of Recommendation Taxonomy (SORT) guidelines for systematic reviews. A detailed overview of these criteria is provided in Table 1.

### 2.4. Search Strategy

A comprehensive literature search was conducted using PubMed and Cochrane Library databases. The following terms were used in the initial search: “pulpotomy”, “deciduous tooth”*, and (“silicates” OR “dental materials”). Medical Subject Heading (MeSH) terms were strategically combined using the Boolean operators “AND” and “OR” to optimize search specificity and sensitivity: “pulpotomy” AND “deciduous tooth” AND (“silicates” OR “glass ionomer cements” OR “resin Cements” OR “resin composites”). This process yielded 359 articles across the consulted databases.

### 2.5. Selection of Studies

The study selection process was conducted in three sequential phases. Initially, titles were screened to exclude clearly irrelevant studies. In the second phase, abstracts of the remaining studies were reviewed to eliminate non-eligible references. A manual search of references was also performed at this stage. In the final phase, full-text articles from remaining studies were evaluated for inclusion. Two independent reviewers (C.-J.M. and M.B.-P.) conducted the selection process. Discrepancies between reviewers were elucidated through discussion until a consensus was reached.

### 2.6. Data Extraction

The following data were extracted from each included study and tabulated: author(s), year of publication, study title, study design, sample size, results, main findings, and conclusions. Reference citations were also recorded. Due to clinical and methodological heterogeneity, a quantitative meta-analysis was not feasible.

### 2.7. Assessment of Risk of Bias

The risk of bias in the included studies was evaluated using the Risk of Bias in Nonrandomized Studies of Interventions (ROBINS-I) tool [20]. The tool assesses potential sources of bias across several domains, including confounding variables, participant selection, intervention classification of interventions, deviations from intended interventions, missing data, measurement of outcomes, and selection of reported results. Two reviewers (C.C. and M.B.) independently evaluated the risk of bias for each study. All discrepancies were addressed through deliberation and mutual agreement.

### 2.8. Analysis of the GRADE Levels of Evidence

The quality of the evidence from the in vivo and in vitro studies was assessed separately using the GRADE system [21]. This evaluation applies the GRADE (Grading of Recommendations, Assessment, Development, and Evaluation) system to two randomized controlled trials (RCTs) on vital pulp therapy in primary molars. Outcomes were assessed independently for each study, focusing on clinical success, radiographic success, restoration performance, and esthetic outcomes, for instance tooth discoloration. The quality of evidence was rated as high, moderate, low, or very low, with concise justifications.

## 3. Results

### 3.1. Study Selection

**Figure 1:**
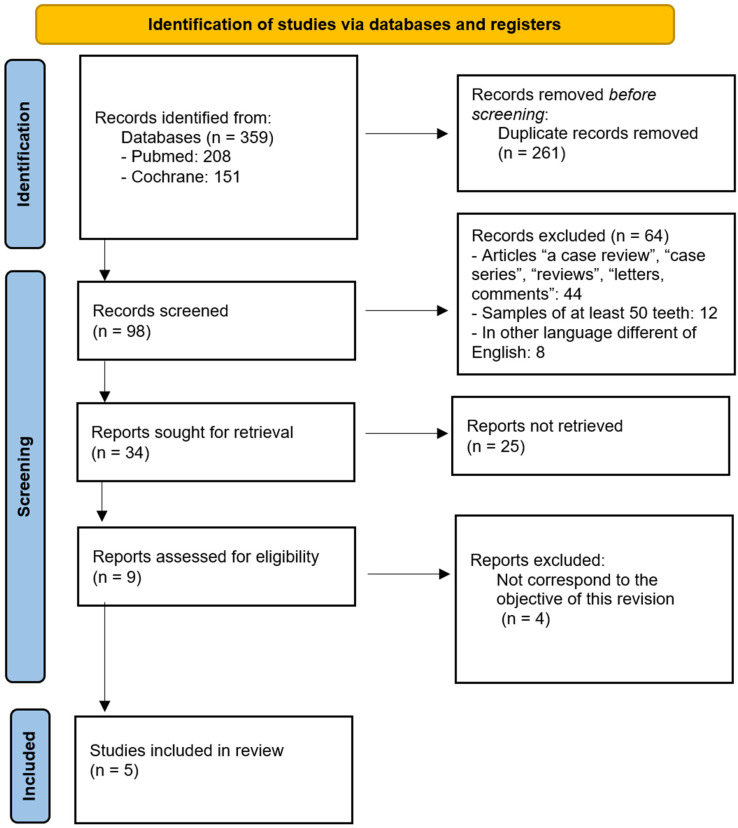
PRISMA flow diagram illustrating the study selection and screening process.

Figure 1 shows the selection process for the included studies. Following the initial database search, 359 records were identified. After applying the defined inclusion criteria, 34 articles were selected for further evaluation. Of these, 25 reports were not retrieved and four were excluded because they do not correspond to the objective of this revision. Ultimately, five studies met all inclusion criteria and were included in the present systematic review [22,23,24,25,26].

### 3.2. Analysis of Included Studies

Table 2 summarizes the key characteristics of the five included studies. The earliest included study was published by Hutcheson et al. in 2012 [22], while the most recent was published by Abdelwahab et al. in 2024 [23]. The studies were conducted in various countries: one in the United States [22], one in Egypt [23], two in Turkey [24,26], and one in the Republic of Korea [25]. Of the five studies, two were in vitro [24,26], while the remaining three were clinical studies in vivo [22,23,24,25]. The sample sizes varied considerably, from 64 teeth [23] to 347 teeth [25]. Biodentine^®^ was used as a pulpotomy material in two studies [24,26], while MTA was used in four studies [22,23,24,25]. In the in vitro study conducted by Topçuoğlu and Topçuoğlu, Biodentine and MTA were used as pulpotomy materials [26].

In the in vitro study by Bolukbasi and Kucukyilmaz [24], the bond strength was evaluated in pulpotomized teeth restored with Biodentine^®^ followed by resin composite or resin-modified glass ionomer cement (RMGIC). Results showed significantly higher bond strength when a resin composite was used. In particular, no cohesive fractures were observed in the composite group, and the adhesive and mixed fracture patterns did not differ significantly between the groups (*p* = 0.331). Scanning electron microscopy revealed complete removal of the dentin barrier layer in samples treated with Biodentine^®^.

Abdelwahab et al. [23] investigated the clinical success of pulpotomized primary molars restored with RMGIC or stainless-steel crowns (SSCs). Their results indicated that RMGIC restorations were significantly associated with a higher risk of failure compared to SSCs (*p* = 0.008). Similarly, Kim et al. [25] reported that direct placement of silver amalgam or RMGIC in MTA-pulpotomized teeth led to a 5.62-fold increased risk of failure compared to SSCs (*p* = 0.002). Hutcheson et al. [22] found that MTA pulpotomies restored with resin composite exhibited more marginal degradation and gray discoloration than those restored with SSCs.

Topçuoğlu and Topçuoğlu [26] compared the fracture resistance of pulpotomized primary molars treated with MTA, Biodentine^®^, and zinc oxide-eugenol (ZOE). The highest resistance to fracture was observed in the unprepared control group (*p* < 0.05), while the positive control group showed the lowest values (*p* < 0.05). Teeth restored with composite after pulpotomy with Biodentine^®^ showed significantly higher fracture resistance than those treated with MTA or ZOE (*p* < 0.05). The most common type of failure in all groups was restorable fracture and no significant differences in failure types were observed (*p* < 0.05).

Table 3 summarizes the risk-of-bias assessment conducted using the ROBINS-I tool for the five included studies. Three studies were classified as high-quality, with a low overall risk of bias [24,25], while two were of moderate quality, presenting a moderate risk of bias [22,23,26]. In one study [22], the domains ‘classification of interventions’ and ‘selection of reported results’ were rated as having a moderate risk of bias; all other domains in that study were judged to have a low risk. Notably, only the study by Kim et al. [25] was rated as having a low risk of bias in the domain of confounding.

Following the assessment of the quality of evidence using the GRADE system [21] for the two in vivo studies, the results are presented in Table 4 and Table 5. Therefore, it is possible to affirm that both studies provide high- to moderate-quality evidence supporting the clinical and radiographic success of MTA and bioactive materials in pulpotomies of primary molars. However, evidence consistently shows that stainless-steel crowns yield superior restoration durability and success compared with esthetic options such as composite or glass ionomer. There is moderate evidence that tooth discoloration commonly occurs with MTA, especially when used with tooth-colored restorations, which may compromise esthetic acceptability. Hence, while bioceramic materials may match MTA in biological performance, the choice of restoration critically impacts long-term outcomes and should be prioritized accordingly in clinical recommendations.

The third in vivo study conducted by Kim et al. in 2021 [25] was analyzed independently due to its retrospective design, which differs methodologically from the randomized controlled trials previously assessed.

Kim et al. [25] evaluated prognostic factors affecting the survival of primary molars after MTA pulpotomy in a retrospective cohort, as shown in Table 6. The overall survival rate received a low GRADE rating because of the study design limitations, lack of a control group, and absence of replication. Survival was significantly better when stainless-steel crowns (SSCs) were used instead of direct fillings, earning a moderate rating for their large effect size despite observational bias. The arch-type comparison (upper vs. lower molars) showed a significant but imprecise difference, also rated low. Caries extending below the CEJ strongly predicted failure, rated moderate due to biological plausibility and large effect size.

In Table 7, it is possible to observe a comparison of the quality of evidence from two in vitro studies using the GRADE system. Bolukbasi et al. (2021) [24] evaluated the bond strength of restorative materials (composite and glass hybrid) after various pulpotomy techniques on primary dentin. Topçuoğlu et al. (2023) [26] assessed the fracture resistance and fracture patterns in pulpotomized primary molars using MTA, Biodentine^®^, or ZOE. All outcomes from both studies were rated as very-low-quality. The main reasons were the in vitro design, limited sample sizes, lack of replication, and indirectness of the results for clinical application. Although the findings suggest that Biodentine^®^ may increase fracture resistance and the Nd:YAG laser may improve bond strength, these results come from controlled laboratory environments that do not fully replicate oral conditions. According to GRADE, the current evidence provides only hypothesis-generating insights. High-quality clinical trials are required before any strong recommendations can be made for clinical practice in pediatric restorative dentistry.

## 4. Discussion

Restoring the morphology and function of primary molars using direct restorative materials poses a significant clinical challenge, particularly in the context of multisurface caries. Increased occlusal forces exerted on large restorations often lead to premature failure. Given the continuous emergence of new adhesive technologies, evaluating their performance against conventional stainless-steel crowns (SSCs) is essential to optimize long-term outcomes and improve the quality of life related to oral health in pediatric patients [27,28]. Currently, SSCs have been considered the ‘gold standard’ for definitive restoration of pulpotomized primary teeth due to their proven reliability and durability [29].

Although SSCs remain the most widely used and preferred restoration in pulpotomized teeth [30], their esthetic limitations and susceptibility to plaque accumulation have driven the search for alternative materials. This clinical demand has catalyzed research into restorative approaches that are not only biologically and mechanically sound but also meet esthetic preferences and prioritize patient needs and preferences. Several standardized evaluation parameters have been proposed to assess restorative success [4]. Ideally, a restoration should mimic the appearance of the natural dentition, be minimally invasive, be efficient in terms of time, and provide sufficient durability until physiological exfoliation [31].

With recent advancements in adhesive dentistry, materials such as glass ionomer cements (GICs) and resin composites have gained increasing attention. However, multiple factors influence the longevity of these restorations, including clinician experience, cavity size, the ability to maintain absolute isolation (for example, with rubber dam), and the anatomical location of the tooth [31].

In the high-quality study by Bolukbasi and Kucukyilmaz [24], the shear bond strength was compared between resin composites and resin-modified glass ionomer cements (RMGICs) in pulpotomized teeth restored over Biodentine^®^. Resin composites demonstrated significantly higher bond strength. This may be attributed to the chemical affinity of adhesive monomers (carboxyl and phosphate groups) with hydroxyapatite crystals, as well as the moderate pH of self-etching primers used in bonding systems [32,33]. Additional studies have confirmed that GICs exhibit inferior dentin adhesion compared with resin composites [34,35], and the application of acidic agents does not appear to enhance the bond strength [36,37].

Although few studies have specifically examined the bond strength of restorative materials after Biodentine^®^ removal, the available data suggest a stronger bond between Biodentine^®^ and resin composites than between Biodentine^®^ and GICs [30,31,32,33,34,35,36,37,38]. This may be due to the unique characteristics of Biodentine^®^ [38], a tricalcium silicate-based cement, which forms a calcium-rich ‘mineral infiltration zone’ on the dentin surface. Diffusion of calcium ions promotes micromechanical interlocking and bioactivity, enhancing the adhesion of restorative materials [39]. MTA, widely studied as a pulpotomy material in primary molars, is well known for its biocompatibility, sealing capacity, high alkalinity, and antimicrobial properties [39]. It also stimulates the release of cytokines by fibroblasts, promoting the formation of hard tissue [40], and has shown regenerative capacity in vital pulp therapy [41]. However, regardless of the pulpotomy material used, the choice of final restoration remains a key factor influencing clinical success.

In the medium-quality study by Topçuoğlu and Topçuoğlu [26], the fracture resistance of pulpotomized primary molars was compared between MTA and Biodentine^®^. Higher resistance was reported in teeth treated with Biodentine^®^, possibly due to its finer and more homogeneous particle size and higher compressive strength compared to MTA [42,43,44].

In this review, two studies [23,24,25] evaluated MTA-treated teeth restored with SSCs or RMGICs. The high-quality study by Kim et al. [25] demonstrated significantly higher failure rates in RMGIC-restored teeth compared to SSCs. This supports the existing literature advocating SSCs as the preferred restoration after pulp therapy due to their ability to maintain a durable coronal seal, an essential factor for long-term clinical success [5,6,7]. Similarly, the study by Abdelwahab et al. [23] reported superior clinical and radiographic success after 12 months in the MTA + SSCs group compared to MTA + RMGICs.

In contrast, Hutcheson et al. [22], in the medium-quality study included in this revision, found that although composite restorations exhibited greater proximal deterioration and discoloration than SSCs, this did not appear to affect overall treatment success within a 12-month follow-up. However, esthetic deterioration raises concerns about long-term function and acceptability. These findings align with those of Sonmez et al. [45], who reported similar results when comparing SSCs and esthetics restorations using an alkasite material (Cention-N). However, the long-term performance and durability of esthetic restorations remain questionable.

The limitations of the studies included in this review must be acknowledged. These include variability in the selection of carious primary molars, which introduces potential confounders, lack of standardized radiographic techniques due to patient age, retrospective study designs, and inconsistencies in treatment protocols. Additionally, in vitro studies often apply forces along the tooth’s long axis, which does not accurately replicate the multidirectional nature of masticatory forces encountered clinically. Therefore, these findings should be interpreted with caution.

Future research should focus on well-designed in vivo clinical trials with standardized protocols, extended follow-up periods, and rigorous confounding variable control. Laboratory studies should also further evaluate the biomechanical behavior of primary teeth restored with emerging materials under clinically simulated loading conditions.

## 5. Conclusions

Considering the results and the limitations of the present systematic review, the following conclusions may be established:

- Regardless of the pulp therapy material used, stainless-steel crowns (SSCs) offer the highest survival rates for pulpotomized primary molars. SSCs should be used to maximize the long-term survival of pulp therapy in children whenever possible;
- According to in vitro studies, Biodentine^®^ may be preferred as a base material in pulpotomized primary teeth, particularly when restoring with composite resins, due to its superior fracture resistance and bonding properties; for pediatric dentists, this indicates that Biodentine^®^ could be the material of choice when high-strength stainless-steel crowns (SSCs) are not used, as it provides sufficient resistance to maintain the integrity until the treated tooth naturally exfoliates;
- Pediatric dental practice is in a state of continuous evolution, not only because of the constant emergence of new materials but also because of the growing esthetic demands of pediatric patients and their caregivers. Replacing stainless-steel crowns with esthetic restorative materials that can offer the same long-term performance in pulpotomized teeth has become a shared goal among patients, dental professionals, and manufacturers alike.

## Figures and Tables

**Table 1:** Inclusion and exclusion criteria.

Inclusion Criteria	Exclusion Criteria
Studies carried out in the last 15 years	Case reports, case series, reviews, letters, and commentaries
Those who evaluated immediate restorations with different materials in pulpotomized teeth	Those which did not correspond to the objective of this review
Papers in English	Pulpotomies performed on permanent teeth or with materials other than MTA or Biodentine^®^
Samples of at least 50 teeth	

**Table 2:** Description of included studies.

Author, Year, and Country	Type of Study	Sample Size	Groups	Success (%)	Fracture Resistance	Shear Bond Strength
Hutcheson et al., 2012 [22], USA	In vivo	74 teeth	MTA + composite, MTA + SSCs	72.3% composite, 87.3% SSCs	Not registered	Not registered
Abdelwahab et al., 2024 [23], Egypt	In vivo	64 teeth	MTA + RMGICs, MTA + SSCs	75% RMGICs, 87.5% SSCs	Not registered	Not registered
Kim et al., 2020 [25], Republic of Korea	In vivo	347 teeth	MTA + RMGICs, MTA + SSCs	76.7% RMGICs, 93.1% SSCs	Not registered	Not registered
Topçuoğlu and Topçuoğlu, 2023 [26], Turkey	In vitro	75 teeth	MTA + composite, Biodentine^®^ + composite	Not registered	334.1 N (MTA), 496.3 N (Biodentine^®^)	Not registered
Bolukbasi and Kucukyilmaz, 2021 [24], Turkey	In vitro	240 teeth	Biodentine^®^ + RMGICs, Biodentine^®^ + composite	Not registered	Not registered	6.70 MPa, 12.60 MPa

MTA: Mineral trioxide aggregate; SSCs: stainless-steel crowns; RMGICs: resin-modified glass ionomer cements; N: newtons; MPa: megapascal.

**Table 3:** Risk-of-bias evaluation according to ROBINS-I.

	Confounding	Selection of Participants	Classification of Interventions	Deviations from Intended Interventions	Missing Data	Measurement of Outcomes	Selection of Reported Results	Overall
Hutcheson et al. (2012) [22]	Moderate	Moderate	Moderate	Moderate	Moderate	Moderate	Moderate	Moderate
Abdelwahab et al. (2024) [23]	Moderate	Low	Low	High	Moderate	Moderate	Low	Moderate
Kim et al. (2020) [25]	Low	Low	Low	Moderate	Low	Low	Low	Low
Topçuoğlu and Topçuoğlu (2023) [26]	Moderate	Moderate	Low	Moderate	High	Moderate	Low	Moderate
Bolukbasi and Kucukyilmaz (2021) [24]	Moderate	Moderate	Low	Low	Moderate	Low	Low	Low

**Table 4:** Hutcheson et al. [22], split-mouth RCT on the restoration type following MTA pulpotomy.

Critical Outcome	GRADE Rating	Justification
Clinical success at 12 months	High	100% success in both groups; split-mouth design, blinded outcome assessment, consistent and precise results
Radiographic success at 12 months	High	100% radiographic success; blinded, calibrated examiners, high precision and applicability
Restoration integrity and durability	Moderate	The composite showed more degradation; not statistically significant; detection bias and small sample size
Tooth discoloration (esthetic acceptability)	Moderate	94% of the composite group showed discoloration; consistent but limited sample; downgraded for imprecision

**Table 5:** Abdelwahab et al. [23], RCT on MTA vs. bioactive pulp-capping materials + SSCs vs. GI restoration.

Critical Outcome	GRADE Rating	Justification
Clinical success at 12 months	Moderate	Robust RCT with good follow-up; high clinical success (>85%), but small sample size and few failures introduce imprecision
Radiographic success at 12 months	Moderate	No significant difference between groups (~70% success); a small sample and few failures introduce uncertainty
Restoration-related failure (SSCs vs. GI)	Moderate	GI restoration significantly increased the failure risk; some detection bias and low event count, but consistent with prior research
Tooth discoloration (esthetic outcome)	Very low	Not directly assessed; theoretical lower risk with BC RRM-F, but no patient data reported

**Table 6:** Kim et al. [25]: retrospective cohort study on prognostic factors after MTA pulpotomy.

Critical Outcome	GRADE Rating	Justification
Overall survival of pulpotomized molars	Low	Retrospective design, moderate sample size, some bias, and no control group; lacks replication
Survival by restoration type (SSCs vs. fillings)	Moderate	Large effect size favoring SSCs; observational data with some bias but directly applicable
Survival by arch type (upper vs. lower molars)	Low	Significant difference, but wide confidence interval and unconfirmed consistency across studies
Survival by caries extension (above vs. below CEJ)	Moderate	Strong association observed; effect biologically plausible but based on one study without randomization

**Table 7:** GRADE evidence, in vitro studies by Bolukbasi [24] and Topçuoğlu [26].

Outcome (Study)	GRADE Quality	Brief Justification
Bond strength—composite (Bolukbasi, 2021) [24]	Very low	Single in vitro study, small sample size, indirect setting, no clinical data or replication
Bond strength—glass hybrid (Bolukbasi, 2021) [24]	Very low	Laboratory-only data, no clinical context, no external validation, and low precision
Fracture resistance (Topçuoğlu, 2023) [26]	Very low	One laboratory study with limited samples, indirect evidence, imprecise results, and no clinical trials
Fracture pattern (Topçuoğlu, 2023) [26]	Very low	Based on simulation, few events, no replication, and lacks applicability to real-world fracture behavior

## Data Availability

The original contributions presented in this study are included in the article. Further inquiries can be directed to the corresponding authors.
